# Predicting compatibility between ferredoxins and the Fe protein of nitrogenase using in silico protein modeling

**DOI:** 10.1002/pro.70509

**Published:** 2026-02-23

**Authors:** Adity Biswas, Katerina Trachtova, Kathryn R. Fixen

**Affiliations:** ^1^ BioTechnology Institute University of Minnesota St. Paul Minnesota USA; ^2^ Department of Plant and Microbial Biology University of Minnesota St. Paul Minnesota USA

**Keywords:** electron transfer, ferredoxin, nitrogen fixation, nitrogenase

## Abstract

Biological nitrogen fixation is the process by which certain bacteria and archaea use the enzyme nitrogenase to reduce atmospheric nitrogen into bioavailable ammonium. Engineering non‐nitrogen‐fixing organisms, like plants, to use nitrogenase could reduce dependency on synthetic fertilizer and mitigate the environmental impacts of industrial fertilizer production. However, nitrogenase activity requires delivery of reducing power by small electron carrying proteins known as ferredoxins and flavodoxins, and successfully engineering nitrogenase into new systems will require a mechanistic understanding of electron delivery by these proteins. Most organisms often have multiple ferredoxins, raising the question of which ferredoxin can support nitrogenase activity. The purpose of this study is to gain insight into how we can predict which ferredoxin is compatible with the Fe protein, the component of nitrogenase that interacts with ferredoxin or flavodoxin. Our in silico protein–protein docking simulations reveal that most ferredoxins and flavodoxins involved in nitrogen fixation have the shortest distance (≤10 Å) between their redox cofactor and the [4Fe‐4S] cluster of the Fe protein. We found shorter cofactor distance contributes to faster intermolecular electron tunneling rates. Bacterial ferredoxins that play a role in nitrogen fixation also exhibit more complementary interactions with the Fe protein than bacterial and plant ferredoxins not involved in this process. Heterologous expression of a set of ferredoxins from both nitrogen‐fixing and non‐nitrogen‐fixing bacteria in the diazotroph *Rhodopseudomonas palustris* supports our model‐derived prediction that shorter distances between the electron‐carrying cofactors favor nitrogenase compatibility. These findings offer a framework to predict and potentially enhance ferredoxin–nitrogenase compatibility, which will help to improve our ability to engineer nitrogen fixation into non‐nitrogen‐fixing organisms like plants.

## 1 INTRODUCTION

Biological nitrogen fixation is the reduction of atmospheric nitrogen gas (N_2_) into bioavailable ammonium (NH_4_^+^) by the oxygen‐sensitive enzyme nitrogenase. Biological nitrogen fixation is a restricted metabolic trait found in specific bacterial and archaeal species and was recently found to occur in a marine alga, *Braarudosphaera bigelowii* (Boyd et al. 2015; Boyd and Peters 2013; Burgess and Lowe 1996; Coale et al. 2024). Historically, limited fixed nitrogen availability constrained crop production until the development of the industrial Haber‐Bosch process, which produces 200 million tons of fertilizers annually to support global agriculture (Nosherwani and Neto 2021; Wyer et al. 2022). However, this process has significant environmental costs, including greenhouse gas emissions and eutrophication from fertilizer runoff (Li et al. 2020; Zhang et al. 2024). One alternative to reduce reliance on synthetic fertilizer involves engineering plants to fix nitrogen using nitrogenase (Santi et al. 2013). Recent advancements in identifying the minimal gene set required for nitrogen fixation and successful expression of nitrogenase components in eukaryotic organelles, such as chloroplasts and mitochondria, highlight significant progress in building a functional nitrogenase in non‐nitrogen‐fixing organisms (Berman et al. 1985; Curatti and Rubio 2014; Dixon et al. 1997; Li et al. 2020; Smanski et al. 2014; Temme et al. 2012; Wang et al. 2013; Zamir et al. 1981).

Nitrogenase is composed of two subunits, a homodimeric NifH subunit known as the iron (Fe) protein and a heterotetrameric NifDK subunit known as the iron‐molybdenum (MoFe) protein (Georgiadis et al. 1992). Over eight electron‐transferring catalytic cycles, the Fe protein hydrolyzes 16 ATP and delivers 8 electrons to the MoFe protein to reduce one molecule of N_2_ to two molecules of ammonia and one molecule of hydrogen. The Fe protein uses electrons donated by low potential protein electron carriers such as ferredoxin or flavodoxin (Braaksma et al. 1982; Watt et al. 1986). Both the Fe protein and the MoFe protein have been successfully expressed in plant and yeast organelles, suggesting that active nitrogenase could potentially be assembled in these compartments (Liu et al. 2025; Ivleva et al. 2016; López‐Torrejón et al. 2016; Payá‐Tormo et al. 2024; Solomon et al. 2024; Wang et al. 2013). However, successful integration of nitrogenase into non‐nitrogen‐fixing organisms, including plants, will also require an understanding of the properties of ferredoxins to deliver electrons to the Fe protein. Previous studies have demonstrated that plant ferredoxins from the chloroplast and root plastid can support some nitrogenase activity in *Escherichia coli* engineered to express nitrogenase (Yang et al. 2017a). However, the molecular underpinnings that led to the variability in nitrogenase activity supported by these ferredoxins remain unclear.

Electron transfer between ferredoxin or flavodoxin and the Fe protein proceeds through multiple steps that include the formation of a transient donor‐acceptor complex, electron tunneling within the complex, and subsequent dissociation. Understanding these steps will be important to find electron carriers that are compatible with the Fe protein or find ways to improve this compatibility in engineered systems. However, many organisms encode multiple ferredoxins and flavodoxins, and even in some diazotrophs the specific electron carriers that interact with the Fe protein are not always known (Campbell et al. 2019; Hanke and Mulo 2013; Poudel et al. 2018). Developing an approach to narrow down which carriers are likely to be compatible with the Fe protein would greatly accelerate efforts to engineer nitrogen fixation into non‐nitrogen‐fixing hosts.

While binding affinity and kinetics are often the focus of electron transfer between proteins, the redox centers must be brought close enough together so that electron tunneling is not rate limiting (Page et al. 2003). Studies on both intra‐ and interprotein electron transfer have shown that natural selection acts on two key parameters for efficient electron tunneling: the redox potentials of the cofactors and the distance separating them (Moser et al. 1992; Moser et al. 2006; Moser et al. 2008; Moser et al. 2010; Moser and Dutton 1992; Page et al. 1999; Tezcan et al. 2001). The reported midpoint potential of the ADP‐bound Fe protein ranges from −415 mV (Morgan et al. 1986) to −470 mV (Braaksma et al. 1982), suggesting its physiological electron donors should have more negative potentials. However, some ferredoxins and flavodoxins supporting nitrogen fixation, such as FdxH from *Anabaena* (*Nostoc*) sp. PCC7120 (*As*FdxH) (−351 mV; Hurley et al. 1997) and NifF of *Klebsiella oxytoca* (*Ko*NifF) (−412 mV; Deistung and Thorneley 1986), have higher potentials than the Fe protein while others, such as Fer1 from *Rhodopseudomonas palustris* (*Rp*Fer1) (−452, −583 mV; Lewis et al. 2024), have very low potentials. One possible explanation for this range of potentials is that higher concentrations of reduced ferredoxins can shift the reaction equilibrium toward reduction of the Fe protein, even when thermodynamically unfavorable (Alleman and Peters 2023). However, this suggests that redox potential alone does not reliably predict compatibility with the Fe protein, and indeed the redox potential of plant ferredoxins tested in a heterologous nitrogen‐fixing system in *E. coli* had no correlation with nitrogenase activity (Yang et al. 2017a).

We therefore asked whether the distance between electron‐carrying cofactors in a docked complex could better predict which ferredoxins or flavodoxins are compatible with the Fe protein. This was motivated by the idea that natural selection has positioned the cofactors of the Fe protein and its cognate electron donors close enough to support rapid electron transfer. To test this, we simulated protein–protein docking models of the Fe protein from our chassis diazotroph *R. palustris* complexed with electron carriers from both nitrogen‐fixing bacteria and non‐nitrogen‐fixing bacteria and plants (Kozakov et al. 2017). We measured the edge‐to‐edge distance between redox cofactors of the partner proteins, and we found most ferredoxins not associated with nitrogen fixation typically dock at distances larger than ferredoxins and flavodoxins involved in nitrogen fixation. The increased distance between the iron–sulfur (FeS) clusters results in slower electron tunneling rates, even when the redox potential of the ferredoxin is lower than −400 mV. We present evidence that the distance between the electron‐carrying cofactors is a better indicator of compatibility between a ferredoxin and the Fe protein than redox potential alone. These findings suggest that shorter distances between FeS clusters of a ferredoxin and the Fe protein could enhance the ability of a plant ferredoxin to support nitrogenase activity.

## 2 RESULTS

### 2.1 Most electron carriers supporting nitrogen fixation have shorter distances (≤10 Å) between their redox cofactor and the FeS cluster of the Fe protein

**FIGURE 1:**
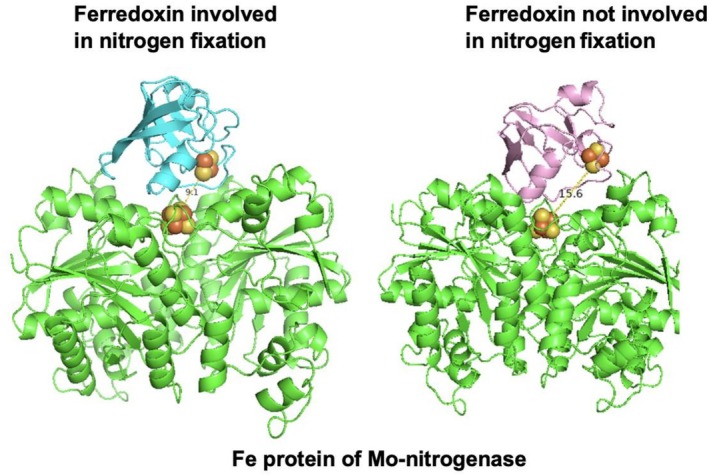
The edge‐to‐edge cofactor distance between a ferredoxin involved in nitrogen fixation and the Fe protein of nitrogenase is shorter compared to a ferredoxin not involved in nitrogen fixation. The cofactor distance between a bacterial ferredoxin that supports nitrogen fixation, FdxH from *Anabaena* (*Nostoc*) sp. PCC7120 (UniProt: P11053), and the Fe protein (WP_011160152) of nitrogenase from *Rhodopseudomonas palustris* falls within 10 Å. While a plant ferredoxin such as FD3 from *Arabidopsis thaliana* (NP_180320.1) shows edge‐to‐edge cofactor distance of 15.6 Å.

We hypothesized that the distance between electron‐carrying cofactors in a flavodoxin or ferredoxin and the Fe protein may be a predictive parameter for assessing their compatibility since the interaction interface has likely been optimized over evolutionary time to bring the electron‐carrying cofactors closer together. We constructed in silico protein–protein docking models using the Fe protein from *R. palustris* paired with either a ferredoxin or flavodoxin from nitrogen‐fixing bacteria (Data S1, Supporting Information), non‐nitrogen‐fixing bacteria (Table S2), and plants (Table S3). In vitro studies demonstrated that ADP‐bound Fe protein interacts with flavodoxin (NifF) in *Azotobacter vinelandii* (Yang et al. 2016). Thus, we simulated the ADP‐bound Fe protein of *R. palustris* using a template‐directed model (PDB: 1FP6) of the ADP‐bound Fe protein of *A. vinelandii* (Jang et al. 2000). We used ClusPro2.0 to create all of our docking models because AI platforms such as AlphaFold 3.0 did not generate the ADP‐bound structure of the Fe protein. We then analyzed these models to assess the role of distance between the electron‐carrying cofactor of ferredoxin or flavodoxin and the [4Fe‐4S] cluster of the Fe protein. We measured the edge‐to‐edge distance, *R* (Å), between the [4Fe‐4S] cluster of ADP‐bound Fe protein and either the FeS cluster in a ferredoxin or the FMN in a flavodoxin (Figure 1).

**FIGURE 2:**
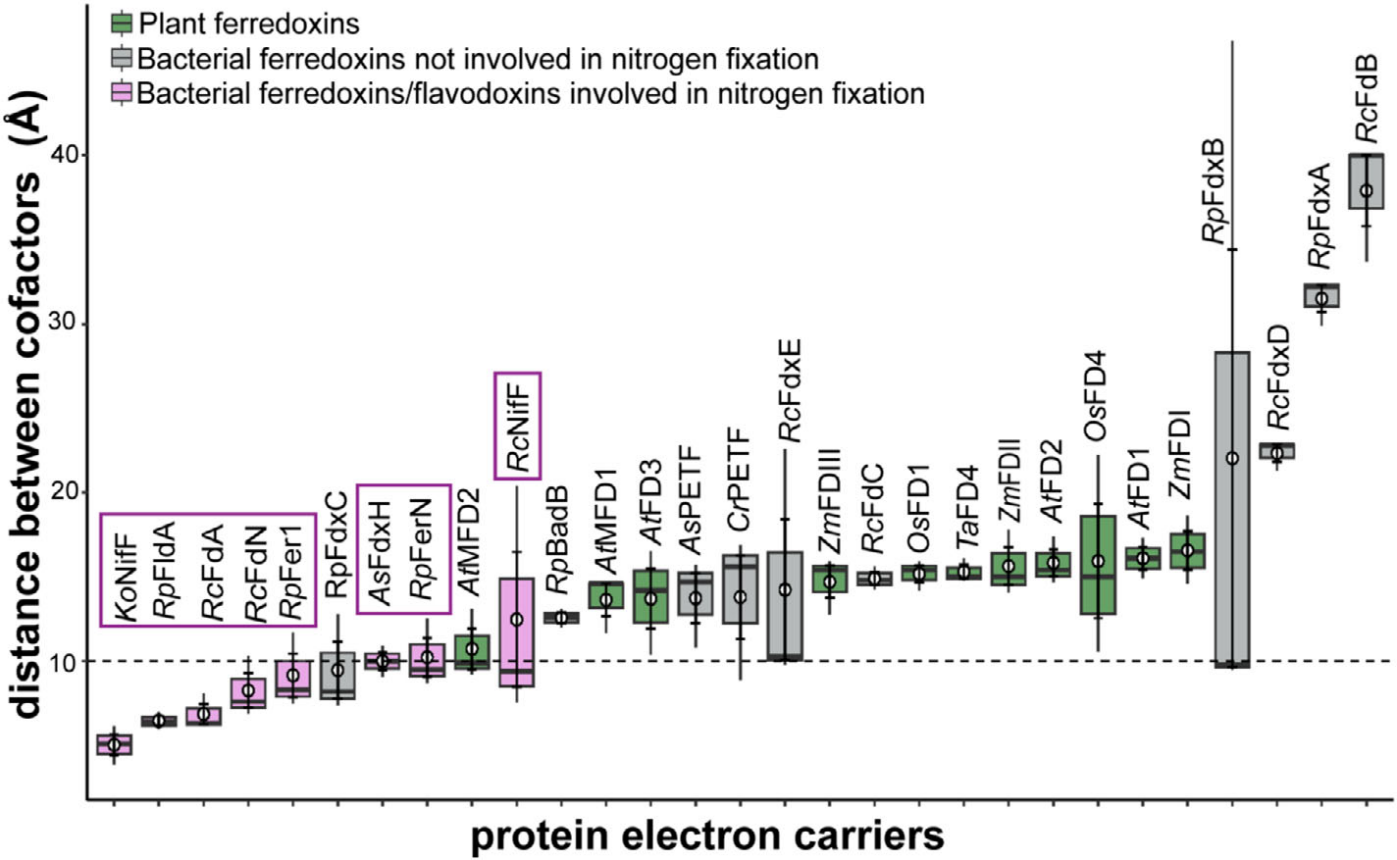
Most electron carriers that support nitrogen fixation have a shorter cofactor distance (≤ 10 Å) from the Fe protein of nitrogenase in docking simulations. Edge‐to‐edge cofactor distance measured in docked complexes with either bacterial ferredoxins/flavodoxins involved in nitrogen fixation (pink), bacterial ferredoxins not involved in nitrogen fixation (gray), or plant ferredoxins (green) and the Fe protein from *R. palustris*. Electron carriers involved in nitrogen fixation include *R. palustris* (*Rp*Fer1, *Rp*FldA, *Rp*FerN), *R. capsulatus* (*Rc*FdN, *Rc*NifF, *Rc*FdA), *K. oxytoca* (*Ko*nifF), and *Anabaena* sp. PCC 7120 (*As*FdxH) highlighted with pink box. The ferredoxins not involved in nitrogen fixation from bacteria include *R. palustris* (*Rp*BadB, *Rp*FdxA, *Rp*FdxB), *R. capsulatus* (*Rc*FdxB, *Rc*FdxC, *Rc*FdxD), *Anabaena* (*Nostoc*) sp. PCC 7120 (*As*PETF), and *C. reinhardtii* (*Cr*PETF). Plant ferredoxins include chloroplast ferredoxins from *A. thaliana* (*At*FD1 and *At*FD2), *Z. mays* (*Zm*FDI and *Zm*FDII), *O. sativa* (*Os*FD1), *T. aestivum* (*Ta*FD4); root plastid ferredoxins from *A. thaliana* (*At*FD3), *Z. mays* (*Zm*FDIII), *O. sativa* (*Os*FD4); and mitochondrial ferredoxins from *A. thaliana* (*At*MFD1, *At*MFD2). Dashed line at 10 Å represents the threshold under which most electron carriers involved in nitrogen fixation fall. Data are from three independent docking simulations. For each electron carrier, the central line in the box shows the median cofactor distance measured, and the edges of the box represent the 25th and 75th percentiles and whiskers extend to the minimum and maximum cofactor distances within 1.5 interquartile ranges. Distance data for each model is listed in Tables S1–S3.

Broadly, the average cofactor distances between the electron carriers supporting nitrogen fixation, including *Rp*Fer1, *Rp*FldA, *Rp*FerN, *Ko*NifF, *Rc*FdN, *Rc*FdA, and *As*FdxH, and the Fe protein had the shortest distances, and the average cofactor distances tended to be shorter than 10 Å, which is consistent with the 6.4 Å previously observed between the flavodoxin NifF and the ADP‐bound Fe protein of *A. vinelandii* (Yang et al. 2016) (Figure 2 and Table S1). This evidence suggests that the shorter average cofactor distance observed for flavodoxins and ferredoxins supporting nitrogen fixation reflects that they can interact with the Fe protein in such a way that the electron‐carrying cofactors are brought closer together.

The distances between cofactors were larger for ferredoxins incapable of supporting nitrogen fixation compared to ferredoxins known to be involved in nitrogen fixation (Figure 2). The average cofactor distance for most bacterial ferredoxins incapable of supporting nitrogen fixation tended to be higher than 10 Å except for FdxC from *R. palustris* (*Rp*FdxC) that had cofactor distances that ranged from 7.5 to 12.8 Å (Figure 2 and Table S2). *Rp*FdxC has not been shown to play a role in nitrogen fixation in *R. palustris*, but its average cofactor distance was less than 10 Å, a distance similar to other ferredoxins involved in nitrogen fixation (Figure 2 and Table S1). *Rp*FdxC has 74% amino acid identity to *Rc*FdA, a ferredoxin from *R. capsulatus*, which is not the main electron donor to nitrogenase in nitrogen‐fixing conditions but acts as an electron donor for nitrogenase in vitro (Jouanneau et al. 2000). However, *Rp*FdxC and *Rc*FdA are required for growth under non‐nitrogen‐fixing conditions, suggesting they serve additional roles (Armengaud et al. 1997; Yang et al. 2017b). These results demonstrate that protein modeling can provide insight into potential interactions between electron carriers and nitrogenase.

There were also cases in which electron carriers produced models with cofactor distances of approximately 10 Å or less and models with a distance greater than 14 Å. These included *Rc*NifF, a known electron donor to the Fe protein, as well as *Rc*FdE and *Rp*FdxB (Figure 2 and Tables S1 and S2). For *Rc*NifF, cross‐linking experiments using NifF from *A. vinelandii* with the Fe protein support the orientation observed in the two models with shorter distances but not in the model with the largest distance (Pence et al. 2017; Yang et al. 2016). Comparable experimental data for *Rp*FdxB and *Rc*FdE are not available, making it unclear which of the models most accurately reflects their native interactions.

The average cofactor distances for most plant ferredoxins found in the chloroplast, root plastid, or mitochondria were greater than 14 Å (Figure 2 and Table S3). The exception being the mitochondrial ferredoxin, *At*MFD2, which had a cofactor distance range of 9.2 to 13.1 Å (Figure 2 and Table S3). We analyzed relative nitrogenase activity for plant ferredoxins reported in an engineered *E. coli* with our measured cofactor distance range and found a significant correlation (*R* ^2^ = 0.644) between cofactor distance and nitrogenase activity reported in Yang et al. (2017a) (Figure S2). These results suggest that modeling cofactor distance provides a useful approach for identifying ferredoxins capable of supporting nitrogenase activity, with shorter cofactor distances corresponding to more effective electron donors to the Fe protein.

### 2.2 Electron carriers supporting nitrogen fixation have the fastest electron tunneling rates

**FIGURE 3:**
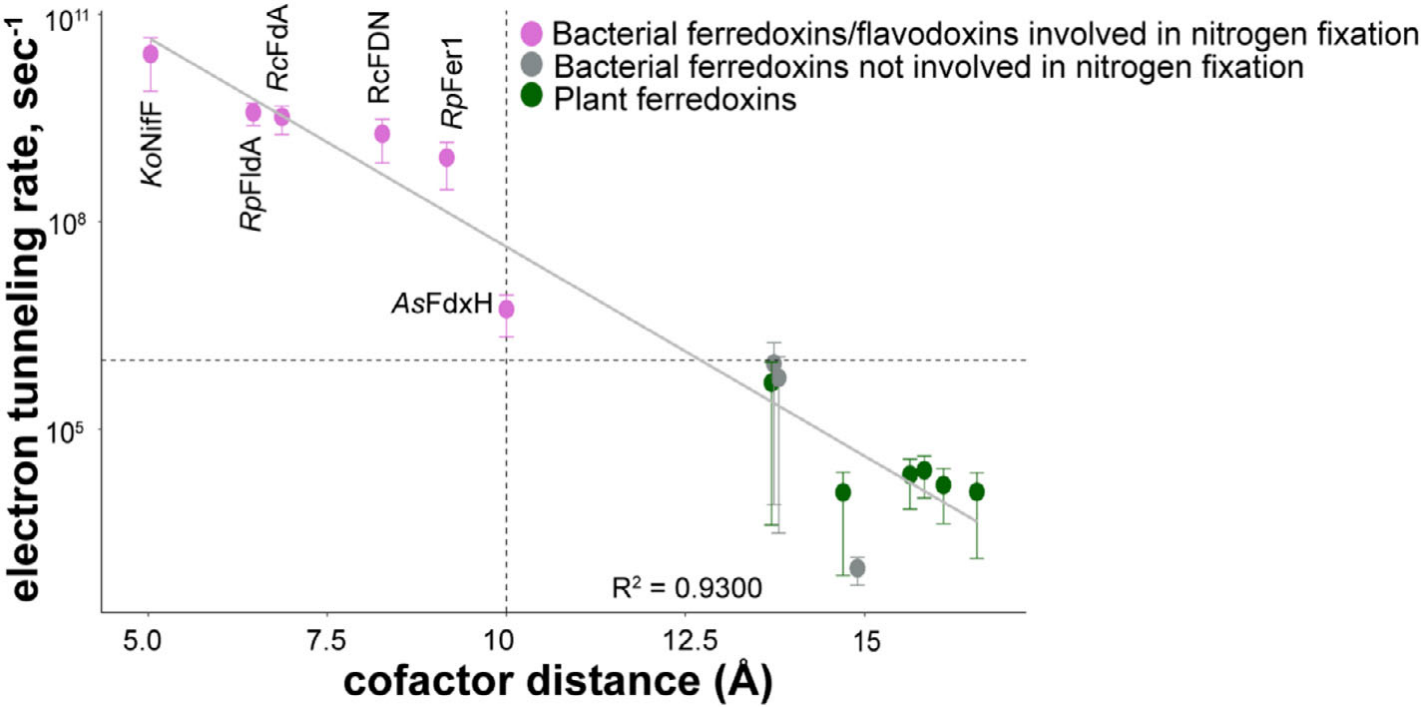
Ferredoxins and flavodoxins involved in nitrogen fixation have faster calculated electron tunneling rates than electron carriers not involved in nitrogen fixation. The mean electron tunneling rate increases as the distance between electron‐carrying cofactors decreases. Ferredoxins and flavodoxins involved in nitrogen fixation (pink circles) had the fastest electron tunneling rates compared to other ferredoxins from bacteria (gray circles) and plants (black circles) that are not involved in nitrogen fixation. Error bars represent one standard deviation from the mean. Dashed line on X‐axis represents a cofactor distance of 10 Å while the dashed line on Y‐ axis represents an electron tunneling rate of 10^6^ s^−1^. Calculated electron tunneling rates used here can be found in Tables S1–S3.

The rate of electron tunneling between the electron‐carrying cofactors depends on both the difference between the redox potentials and the distance between redox cofactors (Page et al. 2003). Electron tunneling rate was calculated for each electron carrier protein and the Fe protein to determine how differences in distance and redox potential impacted electron tunneling between the Fe protein and its electron donor. Two empirical formulas (Equations (1) and (2)) were used to calculate the electron tunneling rate based on either exergonic or endergonic driving force (Moser and Dutton 1992). Ferredoxins and flavodoxins previously shown to be involved in nitrogen fixation had the fastest electron tunneling rates, >10^6^ s^−1^ (Figure 3 and Table S1). This was true even for NifF from *Klebsiella* and FdxH from *Anabaena*, which have a higher midpoint potential than the Fe protein (Deistung and Thorneley 1986; Hurley et al. 1997). Although electron carriers from *Klebsiella* and *Anabaena* share a similar midpoint potential as some of the plant ferredoxins, their electron tunneling rates are much faster because their cofactor distance is shorter. In a natural electron tunneling path, shorter distances between cofactors can enable electron transfer even if electron transfer is thermodynamically unfavorable (Moser et al. 1992; Unciuleac et al. 2004).

In contrast, we found bacterial and plant ferredoxins not involved in nitrogen fixation had much slower electron tunneling rates, ≤10^6^ s^−1^, with an average of 10^4^ s^−1^ (Figure 3 and Tables S2 and S3). Among plant ferredoxins, the root ferredoxin *At*FD3 has the fastest electron tunneling rate, 10^5^ s^−1^. Similar to electron carriers from *Klebsiella* and *Anabaena* described above, *At*Fd3 has a higher redox potential (−337 mV) but has one of the shorter modeled cofactor distances among the plant ferredoxins (13.7 Å) (Pence et al. 2017).

**FIGURE 4:**
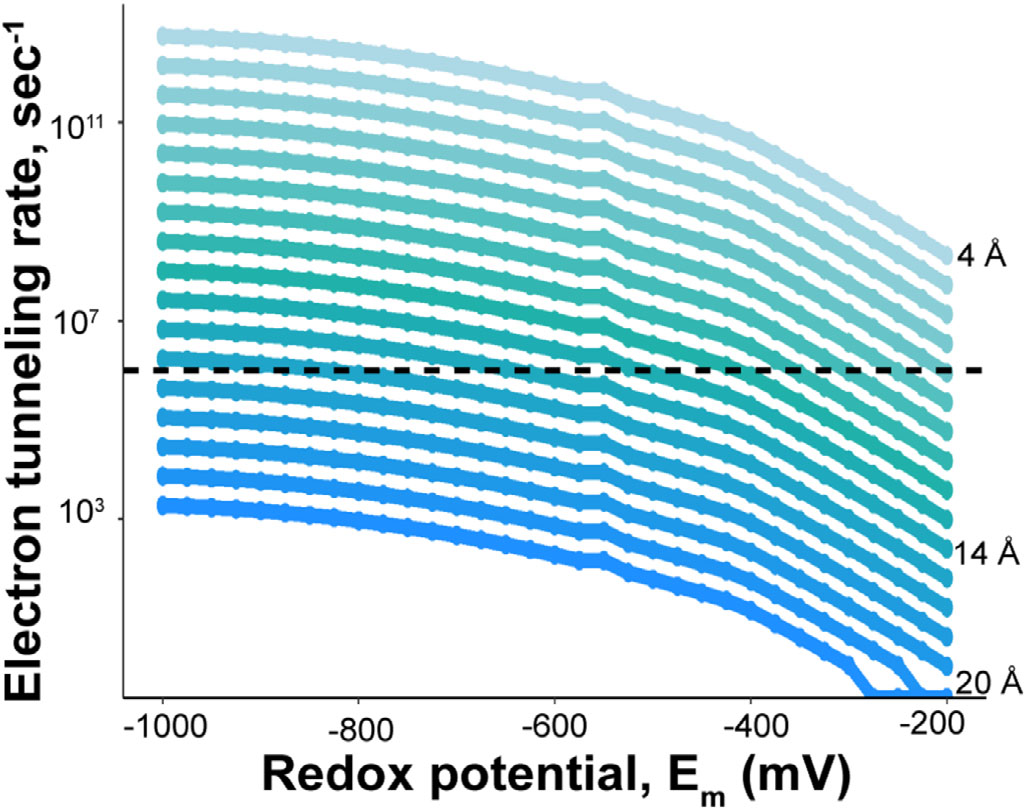
Modeling of calculated electron tunneling rates as a function of cofactor distance and redox potential. The electron tunneling rate at a given potential from −1000 to −200 mV was determined with increasing distance between the cofactors. The shortest distance was 4 Å (top, light blue), and each line represents an increase in distance of 1 Å up to 20 Å (bottom, dark blue line). The dashed line reflects an electron tunneling rate 10^6^ s^−1^.

To investigate how a plant ferredoxin could be engineered to enhance electron transfer to nitrogenase, we modeled electron tunneling rates across a range of redox potentials (from −1000 to −200 mV, in 25 mV increments) and cofactor distances (from 4 to 20 Å, in 1 Å increments) (Figure 4). Assuming that a tunneling rate of at least 10^6^ s^−1^ is necessary to maintain nitrogenase activity since this was the slowest rate calculated for a ferredoxin that supports nitrogen fixation, the cofactor distance between the FeS clusters of the Fe protein and a plant ferredoxin like *Zm*Fd3, which has a redox potential of −321 mV, would need to decrease from 14.7 to 10 Å. However, if the distance between *Zm*Fd3 and the Fe protein stays the same, the redox potential of the *Zm*Fd3 would have to drop to −850 mV to achieve a similar tunneling rate at 10^6^ s^−1^. Since no known [2Fe‐2S] cluster ferredoxin has a redox potential that low, engineering a plant ferredoxin with a shorter cofactor distance may be a more practical strategy to increase the electron tunneling rate from a plant ferredoxin to nitrogenase.

### 2.3 Electrostatic interactions at the protein–protein interface may bring the FeS clusters in a ferredoxin and the Fe protein closer together

**FIGURE 5:**
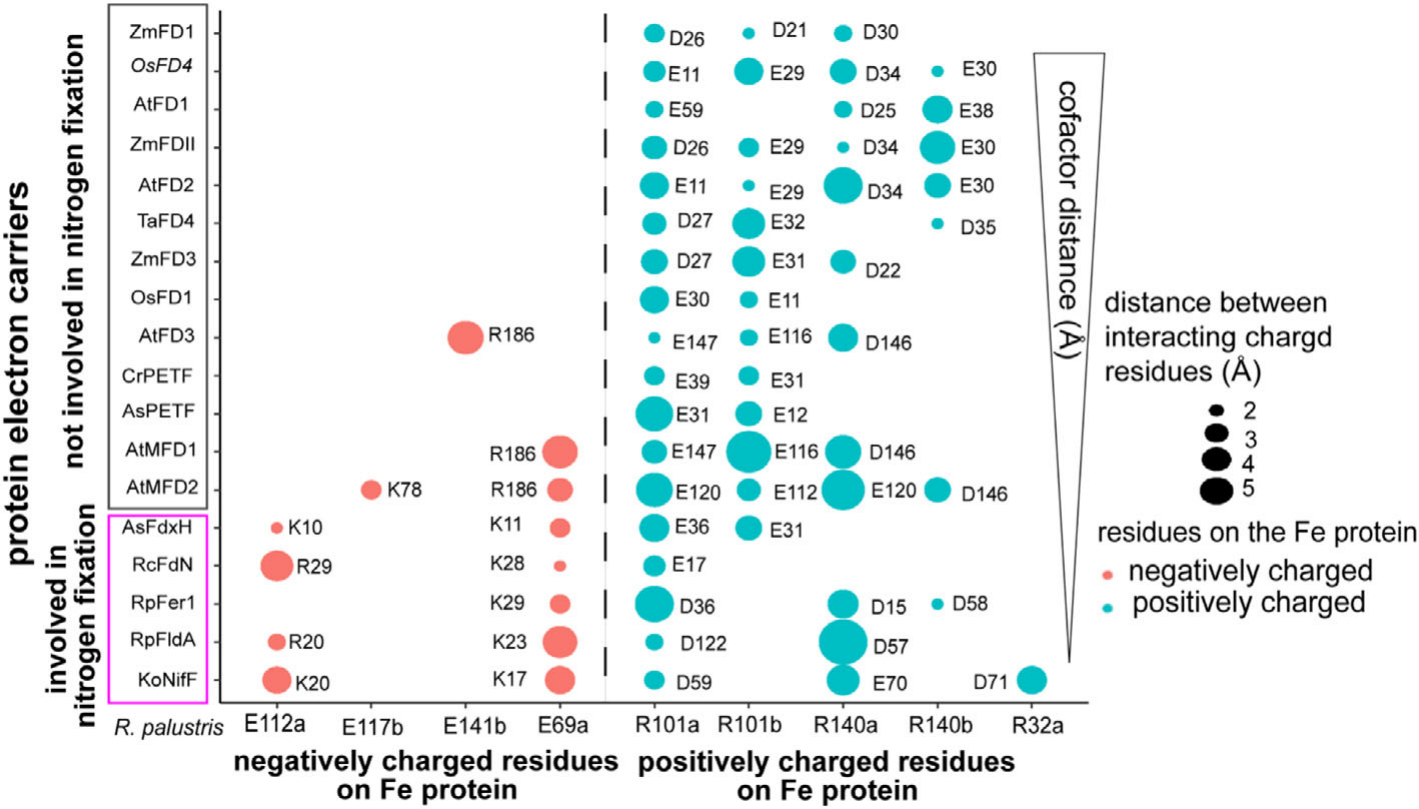
Electron carriers involved in nitrogen fixation have more electrostatic interactions with the Fe protein of nitrogenase. Electrostatic interactions at the docking interface between electron carriers and the Fe protein of nitrogenase arranged by the distance between the [4Fe‐4S] cluster of the Fe protein and the electron transferring cofactor of the ferredoxin or flavodoxin. The interacting charged residues from each electron carrier protein is shown next to a bubble representing the distance between the residue on the protein electron carrier and the corresponding residue on the Fe protein from *R. palustris* shown on the x‐axis. Residues in the protein electron carrier that interact with negatively charged residues of the Fe protein are shown in red and interactions with positive residues of the Fe protein are shown in blue. Because the Fe protein is a homodimer, a and b are used to indicate whether a bond is being made to the same amino acid on the arbitrarily selected chain a or b of the Fe protein dimer.

Our in silico protein modeling suggests a shorter cofactor distance is critical for supporting nitrogenase activity. Therefore, evaluating the docking interface between electron carriers and the Fe protein is essential to identify the factors that influence cofactor distance. Electrostatic interactions at the interface of NifF and the Fe protein in *A. vinelandii* are known to be important for their interaction (Pence et al. 2017). Thus, we analyzed the apparent electrostatic interactions between each modeled electron carrier and the model of the *R. palustris* Fe protein. Charged residues including arginine 100, arginine 140, glutamate 59, and glutamate 104 on the Fe protein were identified in chemical crosslinking and in silico docking experiments when interacting with NifF of *A. vinelandii* (Pence et al. 2017). Our docking models with ferredoxins/flavodoxins supporting nitrogen fixation suggest the corresponding charged residues arginine 101 (R101), arginine 140 (R140), glutamate 112 (E112), and glutamate 69 (E69) on the Fe protein of *R. palustris* are also important to form salt bridge interactions (Figure 5). We performed multiple sequence alignments of homologous Fe proteins from selected diazotrophs from clade I (Nif I), which includes Mo‐nitrogenase from facultative anaerobes; clade II (Nif II), which includes Mo‐nitrogenase from obligate anaerobes; and clade III (Nif III), which includes alternative nitrogenases from both facultative and obligate anaerobes, of the nitrogenase phylogenetic tree (Raymond et al. 2004) (Figure S1). Alignment of these homologous Fe protein sequences indicates R101, R140, E112 are conserved and E69 is less conserved or is replaced with another negatively charged amino acid like an aspartate (D) (Figure S1). This result suggests charged residues and salt‐bridge interactions are a conserved feature of interactions between electron carriers supporting nitrogen fixation and the Fe protein.

To investigate how surface charge of the ferredoxin plays a role in forming salt‐bridge interactions, we compared the individual protein models of ferredoxins supporting nitrogen fixation and ferredoxins not involved in nitrogen fixation. From individual protein models of ferredoxins known to play a role in nitrogen fixation, we found they generally have a cluster of positively charged amino acids and a cluster of negatively charged amino acids on their surface. A similar surface charge pattern on the protein structure of protein electron carriers involved in nitrogen fixation has been observed by others (Pence et al. 2017; Schmitz et al. 1993; Segal et al. 2017). We found the surface charge distribution on ferredoxins supporting nitrogen fixation facilitates the formation of salt‐bridge interactions with the Fe protein (Figure 5). This suggests that the differences in surface charge observed for ferredoxins and flavodoxins involved in nitrogen fixation enable more salt‐bridge interactions, bringing the cofactors closer together.

To further understand the role of charged amino acids, we substituted lysine 10 and 11 with glutamate (K10E) and alanine (K11A) in FdxH from *Anabaena* and did docking simulations with the Fe protein of *R. palustris*. This variant of FdxH (*As*FdxH^K10E,K11A^) resulted in a 31% reduction in in vitro nitrogenase activity compared to wild‐type FdxH (Schmitz et al. 1993). Using *As*FdxH^K10E, K11A^ in docking models also resulted in an increase in the average cofactor distance from 10 to 15.3 Å in our docking models. This result suggests charge compatibility influences the distance between the FeS clusters and may impact nitrogenase activity. Plant ferredoxins have mostly negatively charged amino acids on their protein surface and form fewer salt‐bridge interactions with the Fe protein (Figure 5). While most plant ferredoxins had glutamate and aspartate residues that could interact with R101 and R140 on the Fe protein, they lacked positively charged residues on their surface and tended to form fewer salt‐bridge interactions with E112 and E69 of the Fe protein. In general, our docking simulations indicate that as the number of salt‐bridge interactions increased, the cofactor distance between a ferredoxin and the Fe protein decreased (Figure 5). Our findings also indicate that electron carriers that play a role in nitrogen fixation form more complementary interactions with the Fe protein through salt‐bridge interactions.

### 2.4 Heterologous ferredoxins with cofactor distances around 10 Å from the Fe protein can support nitrogenase activity in *R. palustris*

To test that docking simulations may predict functional compatibility with nitrogenase, we replaced a native ferredoxin required for nitrogen fixation in *R. palustris* with heterologous ferredoxins that varied in their predicted cofactor distances from the Fe protein. To do this, we developed *R. palustris* as an expression platform for testing the effectiveness of heterologous ferredoxins in vivo. First, to ensure reliance on the heterologous ferredoxin, we deleted several other known electron carriers that might participate in delivering electrons to nitrogenase in *R. palustris*: *fldA*, the gene encoding flavodoxin that participates in electron transfer during iron limitation (Fixen et al. 2018), and two 2[4Fe‐4S] ferredoxins, *ferN* and *badB*. Next, we replaced *fer1*, the primary 2[4Fe‐4S] ferredoxin involved in nitrogen fixation in *R. palustris* (Fixen et al. 2018), with genes encoding other 2[4Fe‐4S] ferredoxins from a variety of bacterial species (Table 1). Ferredoxins from *Clostridium pasteurianum* (*Cp*), *Thermotoga maritima* (*Tm*), *E. coli* (*Ec*), and *Chlorobaculum tepidum* (*Ct*) were chosen because they have similar redox potentials to electron carriers involved in nitrogen fixation (Table 1).

**TABLE 1:** Properties of 2[4Fe‐4S] ferredoxins heterologously expressed in *R. palustris* from the *fer1* locus.

Organism and ferredoxin name	Cofactor distance (Å) range	Redox potential (mV)^a^, ^b^	Predicted translation initiation rate^c^
*Clostridium pasteurianum*; *Cp*Fd	8.7, 9.0, 9.1	−380	6.60
*Thermotoga maritima*; *Tm*1175	10.02, 9.7, 11.1	−395, −490	15.53
*Chlorobaculum tepidum*; *Ct*FdII	12.3, 10.2, 10.5	−584	20.07
*Esherichia coli*; *Ec*Fd	7.6, 7.0, 19.7	−418, −675	8.89
*Thermotoga maritima*; *Tm*1815	5.2, 36.0, 37.0	−320, −725	1.48

*Note*: Information on protein sequence and PDB ID for template used for these proteins are in Table S5.

^a^ A single potential indicates two clusters with the same potential.

^b^ The higher redox potential was used to calculate electron tunneling rate for ferredoxins with distinct potentials.

^c^ Translation initiation rate was calculated using the RBS calculator (Salis et al. 2009).

**FIGURE 6:**
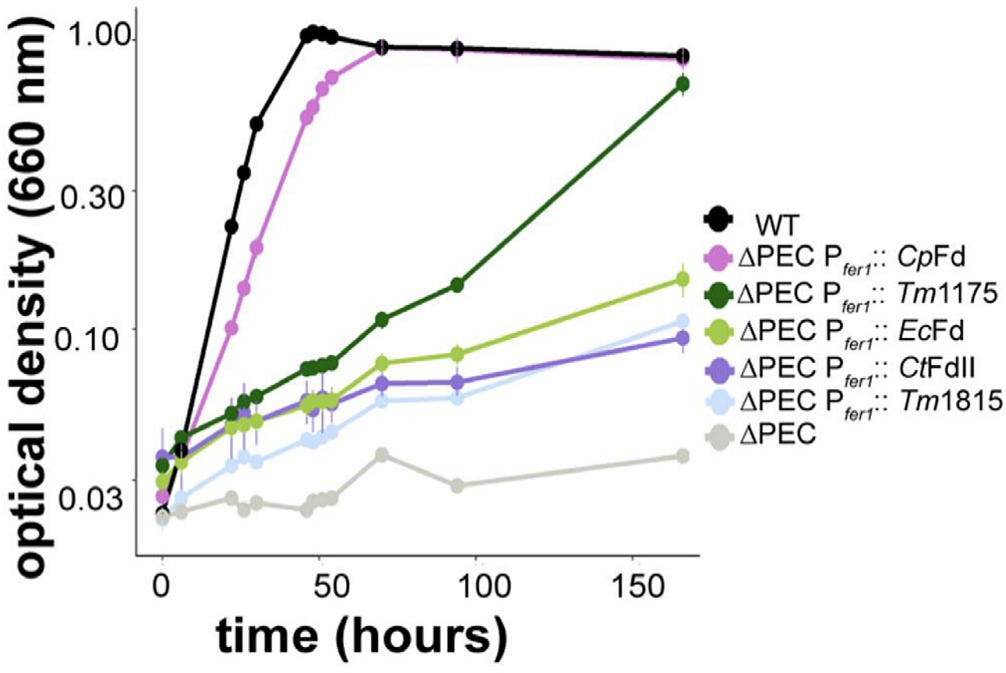
Heterologous expression of a ferredoxin from *C. pasteurianum* or *T. maritima* supports faster growth rates in an *R. palustris* strain lacking native protein electron carriers under nitrogen‐fixing conditions. Growth of *R. palustris* CGA009 (WT), *R. palustris* ∆*fer1* ∆*fldA* ∆*ferN* ∆*badB* (∆PEC); or ∆PEC expressing a heterologous 2[4Fe‐4S] ferredoxin from the *fer1* locus under nitrogen‐fixing conditions, in the light, with 20 mM acetate. The ferredoxins include 2[4Fe‐4S] ferredoxins from *C. pasteurianum* (*Cp*Fd), *T. maritima* (*Tm*1175 and *Tm*1815), *C. tepidum* (*Ct*FdII), and *E. coli* (*Ec*Fd). Growth phenotypes are shown as the average of three biological replicates, and error bars represent one standard deviation from the mean.

These ferredoxins were modeled with the Fe protein to measure the distance between the FeS clusters (Table 1). *Cp*Fd and *Tm*1175 were predicted to have the shortest average cofactor distances (10 Å or less) from the Fe protein, and the remaining bacterial ferredoxins have average distances over 11 Å (Table 1). *R. palustris* expressing *Cp*Fd grew similar to wild‐type *R. palustris*, and *R. palustris* expressing *Tm*1175 had a significantly slower doubling time but still reached high cell densities comparable to wild‐type (Table S6 and Figure 6). *R. palustris* expressing *Ec*Fd, *Ct*Fd, or *Tm*1815 had the slowest doubling times (Table S6 and Figure 6). Despite the reduced growth rate, *R. palustris* expressing *Ec*Fd, *Ct*Fd, or *Tm*1815 grew better than a strain containing in‐frame deletions in *fer1*, *ferN*, *badB*, and *fldA*, indicating they can support some nitrogen fixation (Table S6 and Figure 6).

Since heterologous sequences can impact translation from the same ribosomal binding site, the expected translation initiation rate was calculated using RBS calculator (Salis et al. 2009). We did not see a correlation between the expected translation initiation rate and activity. Both *Tm*1175 and *Ct*Fd are expected to have the highest rates, while *Cp*Fd and *Tm*1815 had the lowest rates, yet *R. palustris* expressing *Cp*Fd or *Tm*1175 had significantly faster doubling times than strains expressing either *Ct*Fd or *Tm*1815 (Table S6). Together, our results indicate that predicted cofactor distance may serve as a useful proxy for identifying ferredoxins that may be more compatible with the Fe protein and may be an important initial step in the selection of electron carriers for nitrogen fixation in synthetic systems.

## 3 DISCUSSION

The purpose of this study was to predict and potentially identify ways to improve the compatibility of a protein electron carrier with the Fe protein of nitrogenase. To achieve this, we simulated interactions between the Fe protein and a variety of electron carriers. We found that ferredoxins and flavodoxins that play a role in nitrogen fixation tended to have shorter distances between their electron‐carrying cofactor and the [4Fe‐4S] cluster in the Fe protein in docking models (Figure 2). While most of the distances fell near or below 10 Å, the absence of experimentally determined structures for these complexes limits our ability to assess the accuracy of these measurements. In this context, these distances are interpreted not as precise structural predictions but as indicators of whether an electron carrier is capable of sampling orientations competent for electron transfer with the Fe protein.

This interpretation follows from the dynamic docking model, which has emerged from studies of other electron transfer systems (Chohan et al. 2001; Leys et al. 2003; Liang et al. 2002; Scanu et al. 2013). Dynamic docking proposes that electron transfer protein partners do not form a single, rigid complex. Instead, they exist as ensembles of many weakly bound orientations, only some of which are positioned appropriately for electron transfer. Forming an ensemble of orientations is thought to be particularly prevalent in transient interactions such as those for electron carriers and their partner proteins (Andrałojć et al. 2017; Blundell and Fernández‐Recio 2006; Crowley and Ubbink 2003; Medina et al. 2008). As a result, whether a given electron carrier can access conformations in which the cofactors come within tunneling range and adopt favorable alignment is important to understand. Under this framework, short cofactor distances in docking models may indicate that an electron carrier can sample conformations that are productive for electron transfer, whereas longer distances may suggest that the electron carrier rarely or never accesses these states.

We also validated that our distance measurements identified electron carriers that interact directly with nitrogenase. In *R. capsulatus*, *Rc*NifF, *Rc*FdB, *Rc*FdC, *Rc*FdD, and *Rc*FdN are all upregulated under nitrogen‐fixing conditions, but only *Rc*NifF and *Rc*FdN are known to donate electrons to nitrogenase while the other ferredoxins are implicated in other functions (Addison et al. 2025; Armengaud et al. 1994; Jouanneau et al. 1993; Jouanneau et al. 1995; Jouanneau et al. 2000; Yakunin et al. 1993). Of these electron carriers, *Rc*NifF and *Rc*FdN also exhibited the shortest modeled distances between their cofactor and the [4Fe‐4S] cluster in the Fe protein. A similar pattern was observed for electron carriers from *R. palustris*, where only the electron carriers *Rp*NifF, *Rp*FdxB, *Rp*FerN, and *Rp*Fer1 are upregulated under nitrogen‐fixing conditions (Oda et al. 2005). Among these electron carriers, only *Rp*NifF, *Rp*FerN, and *Rp*Fer1 play a role in electron transfer to nitrogenase (Fixen et al. 2018). Docking models with *Rp*NifF, *Rp*FerN, and *Rp*Fer1 and the Fe protein all have cofactor distances close to 10 Å or less, while docking models with *Rp*FdxB and the Fe protein have an average cofactor distance of 22.03 Å, well outside the range for efficient electron tunneling.

Moreover, this approach also suggested distinct roles for less‐characterized electron carriers. For example, although both *Rc*FdN and *Rc*FdC are required for growth under nitrogen‐fixing conditions, *Rc*FdC likely does not act as an electron donor for nitrogenase because of its high redox potential (around −285 mV) (Addison et al. 2025; Jouanneau et al. 2000). The greater distance between the [2Fe‐2S] cluster in *Rc*FdC and the [4Fe‐4S] cluster in the Fe protein supports an alternative role for *Rc*FdC, perhaps as the electron donor for FprA, the nitrogen fixation‐related flavoprotein, as has been previously proposed (Figure 2 and Table S2) (Jouanneau et al. 2000). Interestingly, *Rc*FdA and its homolog *Rp*FdxC have distances less than 10 Å in docking models with the Fe protein, and *Rc*FdA can act as an electron donor to nitrogenase in vitro (Yakunin and Gogotov 1983). However, neither protein has been linked to nitrogen fixation in vivo, likely because they are not upregulated under these conditions and have roles outside of nitrogen fixation (Yakunin and Gogotov 1983; Yang et al. 2017b). Although these carriers can in principle access productive orientations for electron transfer with the Fe protein, their regulation, not their structural compatibility, likely restricts their in vivo roles. Using modeled cofactor distances may be particularly useful in diazotrophs, like *Methanosarcina acetivorans* and *Methanococcus maripaludis*, where it is still unclear what electron carriers are involved in electron transfer to nitrogenase.

To gain insight into what factors enable a shorter distance between the cofactors, we focused on electrostatic interactions. Electrostatics play an important role in an encounter complex by stabilizing docking and influencing the alignment of redox cofactors once the proteins associate (Ubbink 2009). Additionally, experimental evidence suggests that electrostatic interactions are important for the electron carrier‐Fe protein interaction (Jacquot et al. 1997; Schmitz et al. 1993; Vickery 1997; Yan et al. 2013). We found that shorter cofactor distances tended to occur in complexes with more electrostatic interactions (Figure 5). Moreover, a variant of FdxH engineered to reduce electrostatic contacts exhibited an increase in cofactor distance from 10 to 15 Å, suggesting that specific charge–charge interactions help bias the docking complexes toward orientations that are productive for electron tunneling. This suggests that electrostatic interactions could help shift the orientation of the ferredoxin such that the cofactors reside within the ~14 Å limit required for efficient tunneling.

Although we see a correlation between cofactor distance and nitrogenase activity reported in Yang et al. (2017a), it is still unclear how slower tunneling will impact nitrogenase activity. Interprotein electron tunneling exhibits an exponential dependence on distance (Tezcan et al. 2001), and our models indicate that the larger cofactor separations in plant ferredoxins should substantially slow tunneling. Yet even the slowest tunneling rate we estimated likely exceeds the dissociation rate of the two proteins and the rate of P_i_ release by the Fe protein—the established rate‐limiting step of the nitrogenase cycle (Yang et al. 2016). Overall electron delivery is more likely constrained by association and dissociation of the Fe protein and its electron donor than by the tunneling step itself. However, the strongest binding conformation does not always place the cofactors close together, and additional diffusive motions may be needed to sample conformations that align the cofactors (Andrałojć et al. 2017; Blundell and Fernández‐Recio 2006; Crowley and Ubbink 2003; Medina et al. 2008). As a result, electron transfer rates can be much slower than the tunneling rates predicted from a docked complex. Enhancing electrostatic interactions between a plant ferredoxin and the Fe protein could enable orientations that bring the cofactors closer together, increasing the likelihood of productive electron transfer—a consideration that may be especially important for ferredoxins with higher redox potentials, where uphill electron transfer would be even slower.

If cofactor distance is a predictor for whether an electron carrier can achieve productive electron transfer orientations with the Fe protein, then cofactor distance may be an informative first filter to predict which electron carriers will be more compatible with the Fe protein. We found that the two ferredoxins with the shortest modeled distances, *Cp*Fd and *Tm*1175, supported faster growth of *R. palustris* compared to other strains expressing *Ec*Fd, *Ct*Fd, or *Tm*1815, enabling these strains to grow to high cell densities under nitrogen‐fixing conditions. *C. pasteurianum* is a known diazotroph, and *Cp*Fd was the first ferredoxin shown to deliver electrons to nitrogenase, and our result confirms that this ferredoxin can support nitrogenase activity (Mortenson et al. 1963). In contrast, *T. maritima* lacks the genes for nitrogen fixation, and the physiological role of *Tm*1175 is unknown. However, *Tm*1175 is able to support in vitro activity of *T. maritima* MiaB, a radical S‐adenosylmethionine methylthiotransferase (Arcinas et al. 2019). Our findings therefore suggest that proximity of redox cofactors identifies both known and previously unrecognized electron donors capable of supporting nitrogenase.

While cofactor distance appears to be a useful predictor of compatibility, additional factors clearly modulate the ability of heterologous ferredoxins to support nitrogenase. Several studies have shown that *Ec*Fd can support nitrogenase activity in an engineered *E. coli*, albeit at lower levels than when an electron carrier involved in nitrogen fixation is expressed (Liu et al. 2025; Ito et al. 2024; Li et al. 2016). In *R. palustris*, we see that the presence of *Ec*Fd is only slightly better than a strain in which electron transfer to Fe protein is abolished. It remains unclear whether this poor performance reflects weaker compatibility with the Fe protein or other limitations, such as insufficient protein abundance, absence of a specific ferredoxin‐reducing enzyme, or suboptimal FeS‐cluster occupancy. Even so, using cofactor distance as an initial screen could help prioritize electron carriers for experimental testing when engineering nitrogen fixation into new hosts.

## 4 MATERIALS AND METHODS

### 4.1 Protein models of ADP‐bound Fe protein and electron carrier proteins

A structural model of ADP‐bound Fe protein was built using the protein sequence from *R. palustris* (Table S4). Initially, AlphaFold 3.0 was used to predict the Fe protein structure, but AlphaFold 3.0 was unable to generate the ADP‐bound conformation for the Fe protein homodimer. Therefore, we used RoseTTAFold to generate a single monomer of the Fe protein and used the ADP‐bound Fe protein structure from *A. vinelandii* (PDB entry 1FP6) as a template to generate the ADP‐bound homodimer and incorporate the [4Fe‐4S] cluster in PyMOL (v.3.0.3) (Baek et al. 2021; Schrödinger, LLC 2015a; Schrödinger, LLC 2015b; Schrödinger, LLC 2015c). The structure of each bacterial and eukaryotic ferredoxin was generated using AlphaFold 2.0 in UCSF ChimeraX, and the PDB structures used to situate FeS clusters or flavin cofactors are shown in Tables S1–S3 (Pettersen et al. 2021). The electrostatic potential of the surface of each ferredoxin was modeled using the Adaptive Poisson‐Boltzmann Solver (APBS) computational tool in PyMOL (Unni et al. 2011) (Armengaud et al. 1994).

### 4.2 Docking models of bacterial ferredoxins/flavodoxins and plant ferredoxins with NifH

In silico protein–protein docking models were prepared using the computational docking program ClusPro 2.0 using their default settings (Kozakov et al. 2017). All docking simulations were prepared using the model of the *R. palustris* ADP‐bound Fe protein described above as the receptor and the ferredoxin or flavodoxin as a ligand. To measure the edge‐to‐edge distance between the Fe atom of cofactor in the ferredoxin or FMN of flavodoxin and the Fe atom of the [4Fe‐4S] cluster of the Fe protein in PyMOL. For this analysis, the top three docking models that ClusPro 2.0 generated were selected for further analysis. All docking complexes used can be accessed under the DOI: https://doi.org/10.6084/m9.figshare.30401095.v1. The edge‐to‐edge cofactor distance range between cofactors, *R* (Å), of partner proteins using these models is represented in Figure 2 and Tables S1–S3.

### 4.3 Electron tunneling rate calculations

Electron tunneling rate refers to the frequency or probability at which an electron can transfer through a potential barrier. Factors that affect electron tunneling include driving force (∆G^0^, mV) which is the difference between redox potentials of a ferredoxin and the Fe protein, and the edge‐to‐edge cofactor distance, *R* (Å) (Moser et al. 2006). To calculate the electron tunneling rate (s^−1^) from a ferredoxin or flavodoxin to ADP‐bound Fe protein, ∆G^0^ was calculated using the redox potentials previously determined (see references in Tables S1–S3) and the edge‐to‐edge cofactor distance that we measured using protein–protein docking models.

The electron tunneling rate (s^−1^) when ∆G^0^ was exergonic was calculated using Equation (1) (Page et al. 1999),

(1) $${\mathrm{Log}}_{10}K_{\mathrm{et}}^{\mathrm{ex}}=13.0-\left(1.2-0.8\rho \right)\left(R-3.6\right)-3.1{\left(\Delta G+\lambda \right)}^{2}/\lambda .$$

The electron tunneling rate (s^−1^) when ∆*G* ^0^ was endergonic was calculated using Equation (2) (Page et al. 1999),

(2) $${\mathrm{Log}}_{10}K_{\mathrm{et}}^{\mathrm{en}}=\begin{array}{l} 13.0-\left(1.2-0.8\rho \right)\left(R-3.6\right)-3.1(-\Delta G\\ {+\;\lambda )}^{2}/\lambda -\Delta G/0.06. \end{array}$$

To calculate the electron tunneling rate (s^−1^), the reorganization energy (*λ*) used was 0.65 eV, and the packing density (𝜌) proposed to account for protein heterogeneity is 0.76 (Moser et al. 2006). The reorganization energy (*λ*) is the energy required to disrupt the equilibrium of the nuclear geometry of the partner proteins. The reorganization energies for tunneling electrons within proteins range between 0.6 and 0.9 eV in an aqueous phase, with an average value of 0.65 eV proposed for the reorganization energy within a cellular environment (Moser et al. 2006). The calculated electron tunneling rates for each electron carrier are shown in Figure 3a and Tables S1–S3.

## AUTHOR CONTRIBUTIONS

**Adity Biswas:** Writing – original draft; validation; visualization; writing – review and editing; formal analysis; data curation; investigation; methodology. **Katerina Trachtova:** Visualization; validation; investigation. **Kathryn R. Fixen:** Conceptualization; funding acquisition; writing – review and editing; project administration; supervision; resources.

## Supporting information


**Data S1.** Supporting Information.

## Data Availability

The data that support the findings of this study are openly available in Figshare at https://doi.org/10.6084/m9.figshare.30401095.v1.
