# Supplementary material for: Predicting compatibility between ferredoxins and the Fe protein of nitrogenase using in silico protein modeling
Source: Protein Sci. 2026 Feb 23;35(3):e70509. doi: 10.1002/pro.70509 (PMC12929197; doi:10.1002/pro.70509)
Supplement: Supplementary file 1 — Data S1. Supporting Information. [file PRO-35-e70509-s001.docx]

**Supplemental material and methods**

*Growth of R. palustris and its mutants*

Strains, plasmids, and primers are listed in Supplemental Table 6. All cultures of *R. palustris* were grown under anoxic conditions in sealed culture tubes with a headspace containing 97.5% N_2_ and 2.5% H_2_, with oxygen levels kept below 10 ppm. The cultures were maintained under photoheterotrophic conditions, using a 60 W incandescent bulb placed 5.5 inches from the tubes, providing a light intensity of 30 µmol photons m^-^² s⁻¹, as previously described in [1]. *R. palustris* strains were initially grown in minimal mineral medium [2], supplemented with 20 mM acetate and 0.1% yeast extract as carbon sources. Once cultures reached an optical density at 660 nm (OD_660_) of 1.1, they were diluted 1:100 into nitrogen-fixing medium, which is minimal mineral medium with no ammonium sulfate added and N_2_ as the only nitrogen source, with 20 mM acetate. The cells were allowed to undergo several doublings to acclimate the cells to nitrogen-fixing conditions and dilute any ammonium carried over from transfer. After the acclimation, cultures were used to inoculate fresh nitrogen-fixing medium. Growth was monitored over time by measuring optical density at 660 nm.

*Genetic manipulation of R. palustris*

In-frame deletion constructs of *fer1* (*rpa4631*), *fldA* (*rpa2117*), *ferN* (*rpa4629*), and *badB* (*rpa0662*) were prepared by PCR amplified fragments that included 1 kb of upstream of the start codon and 1 kb downstream of the stop codon. Genomic DNA from *R. palustris* CGA009 served as the template for amplification using Phusion High-Fidelity DNA polymerase (New England Biolabs). The two 1-kb fragments for each gene were ligated into pJQ200SK vector using *E. coli* DH5⍺-mediated assembly [3]. All plasmids were introduced into *R. palustris* by conjugation with *E. coli* S17-1, and double-crossover recombination events for gene deletions and screened using a previously described method [4]. The presence of deletions was confirmed by PCR.

Bacterial ferredoxin genes from *C. pasteurianum* (*Cp*), *T. maritima* (*Tm*), *C. tepidum* (*Ct*), and *E. coli* (*Ec*) were codon optimized for *R. palustris* using JAVA Codon Adaptation Tool (<https://www.jcat.de>) [5]. Replacement of *fer1* (*rpa4631*) with the bacterial ferredoxin genes was carried out by constructing a plasmid containing 25bp upstream of the *fer1* start codon and downstream of *fer1* stop codon fused to either side of the bacterial ferredoxin gene and ligated into pJQ200SK vector using *E. coli* DH5⍺-mediated assembly technique [3]. All plasmids were introduced into *R. palustris* by conjugation with *E. coli* S17-1, and double-crossover recombination events for gene deletions or allelic exchange were selected and screened using a previously described method [4]. Integration of bacterial ferredoxin genes was confirmed by colony PCR followed by Sanger sequencing. Strains were also validated using whole genome sequencing (SeqCenter).

**Supplemental Table 1. Nitrogen-fixing bacterial electron carriers used in this study.**

| **Bacterial ferredoxins/ flavodoxins** | **NCBI or UniProt accession number** | **PDB ID for template ^b^** | **cofactor distances from the top three docking complex (Å)^c^** | **Redox potentials (mV)** | **Calculated electron tunneling rate (sec^-1^) ^d^** |
| --- | --- | --- | --- | --- | --- |
| *Rp*Fer1 | P00207 | 2FGO [6] | 11.7, 8.3, 7.5 | -452,-583 [7] | 8.5 x 10^8^ |
| *Rp*FldA | Q6N7Y7 | 8V2Y [8] | 7.0, 6.4, 6.0 | -450 [8] | 3.8 x 10^9^ |
| *Rp*FerN | Q6N0Y0 | 1RGV [9] | 9.5, 8.7, 12.5 | ND^e^ | ND |
| *As*FdxH | P11053 | 1FRD [10] | 9.1, 10.0, 10.9 | -351 [11] | 5.4 x 10^6^ |
| *Ko*NifF | WP_004138775.1 | 1YOB [12] | 5.1, 6.1, 3.9 | -412 [13] | 2.7 x 10^10^ |
| *Rc*FdN | D5ARY6 | 1CLF [14] | 6.8, 7.2, 10.3 | -490 [15] | 1.87 x10^9^ |
| *Rc*FdA | D5AP15 | 6FD1 [16] | 6.3, 6.2, 8.1 | -419 [17] | 3.28 x10^9^ |
| *Rc*NifF | P52967 | 2WC1 [18] | 7.6, 9.4, 20.4 | -487 | 5.45 x10^8^ |

^a^ *Rhodopseudomonas palustris* (*Rp*), *Anabaena (Nostoc) sp.* PCC 7120 (*As*), *Klebsiella oxytoca* (*Ko*), *Rhodobacter capsulatus* (*Rc*).

^b^PDB template was selected based on the structural homology of proteins suggest by Phyre2[19]

^c^the edge-to-edge distance between the electron-carrying cofactor in the ferredoxin or flavodoxin and the [4Fe-4S] cluster in the Fe protein.

^d^ calculated from Eq. 1 and Eq. 2

^e^ND, not determined

**Supplemental Table 2. Non-nitrogen-fixing bacterial ferredoxins used in this study.**

| **Bacterial ferredoxins^a^** | **NCBI or UniProt accession number** | **PDB ID for template ^b^** | **Average cofactor distance (Å)^c^** | **Redox potentials (mV)** | **Calculated electron tunneling rate (sec^-1^) ^d^** |
| --- | --- | --- | --- | --- | --- |
| *Rp*BadB | Q6NC12 | 1RGV [9] | 12.6, 13.1, 12.0 | ND^e^ | ND |
| *Rp*FdxA | Q6N917 | 4ID8 [20] | 32.4, 29.9, 32.2 | ND | ND |
| *Rp*FdxB | Q6N0Z7 | 7QV7 [21] | 9.5, 9.8, 46.8 | ND | ND |
| *Rp*FdxC | Q6NCI3 | 1BQC [22] | 8.2, 7.4, 12.8 | ND | ND |
| *As*PetF | P0A3C8 | 1CZP [23] | 15.7, 10.8, 14.7 | -384 [11] | 8.9 x 10^5^ |
| *Cr*PetF | XP_001692808.1 | 1AWD [24] | 8.9, 10.8, 14.7 | -321 | 5.5 x 10^5^ |
| *Rc*FdC | D5ARY7 | 1FRR [26] | 14.3, 15.6, 14.8 | -285±10 | 9.81 x10^2^ |
| *Rc*FdB | D5ARX7 | 7QV7 [21] | 33.7, 40.0, 40.0 | ND | ND |
| *Rc*FdD | D5ANI4 | 8RHO [27] | 22.8, 22.9, 21.3 | ND | ND |
| *Rc*FdE | P80306 | 1E9M [28] | 22.6, 9.8, 10.3 | ND | ND |

^a^ *Rhodopseudomonas palustris* (*Rp*), *Anabaena (Nostoc) sp.* PCC 7120 (*As*), *Klebsiella oxytoca* (*Ko*), *Rhodobacter capsulatus* (*Rc*), *Chlamydomonas reinhardtii* (*Cr*).

^b^PDB template was selected based on the structural homology of proteins suggest by Phyre2 [19]

^c^ the edge-to-edge distance between the electron-carrying cofactor in the ferredoxin or flavodoxin and the [4Fe-4S] cluster in the Fe protein.

^d^ calculated from Eq. 1 and Eq. 2

^e^ND, not determined

**Supplemental Table 3. Plant ferredoxins used in this study.**

| **Plant ferredoxins^a^** | **NCBI or UniProt accession number** | **PDB ID for template ^b^** | **Average edge to edge cofactor distance (Å)^c^** | **Redox potentials (mV)** | **Calculated electron tunneling rate (sec-^1^) ^d^** |
| --- | --- | --- | --- | --- | --- |
| *Zm*FDI | P27787.1 | 5H57 [29] | 18.6, 14.6, 16.5 | -423 [6] | 1.3 x 10^4^ |
| *Zm*FDII | O80429.1 |  | 14.1, 15.0, 17.8 | -406 [30] | 2.2 x 10^4^ |
| *Zm*FDIII | NP_001105346.1 |  | 15.4, 15.9, 12.8 | -321 [31] | 1.2 x 10^4^ |
| *At*FD1 | NP_172565.1 | 4ZHO [32] | 14.9, 16.1, 17.3 | -425 [33] | 1.6 x 10^4^ |
| *At*FD2 | NP_176291.1 |  | 17.4, 15.4, 14.7 | -433 [33] | 2.6 x 10^4^ |
| *At*FD3 | NP_180320.1 |  | 10.4, 16.5, 14.2 | -337 [33] | 4.7 x 10^5^ |
| *At*MFD1 | NP_001329852 | 5UJ5 [34] | 11.7, 14.6, 14.6 | ND^e^ | ND |
| *At*MFD2 | NP_001031685 | 2WLB [35] | 13.1, 9.9, 9.2 | ND | ND |
| *Os*FD1 | NP_001390240.1 | 1GAQ [36] | 14.2, 15.9, 15.4 | ND | ND |
| *Os*FD4 | XP_066166407.1 | 1CZP [23] | 22.2, 15.0, 10.6 | ND | ND |
| *Ta*FD4 | NP_001413721.1 | 1GAQ [36] | 14.8, 15.0, 16.1 | ND | ND |

^a^ *Arabidopsis thaliana* (*At*), *Zea mays* (*Zm*), *Triticum aestivum* (*Ta, Ta*Fd4), *Oryza sativa* (*Os*).

^b^PDB template was selected based on the structural homology of proteins suggest by Phyre2[19]

^c^ the edge-to-edge distance between the electron-carrying cofactor in the ferredoxin or flavodoxin and the [4Fe-4S] cluster in the Fe protein.

^d^ calculated from Eq. 1 and Eq. 2

^e^ND, not determined

**Supplemental Table 4. NifH sequences used for multiple sequence alignment in this study**

| **Organism** | **Fe protein** | **NCBI or UniProt accession number** |
| --- | --- | --- |
| *Rhodopseudomonas palustris* | NifH | WP_011160152 |
| *Azotobacter vinelandii* | NifH | WP_012698831.1 |
| *Anabaena PCC 7120* | NifH | AAB65801.1 |
| *Klebsiella pneumoniae* | NifH | P00458 |
| *Rhodospirillum rubrum* | NifH | WP_011388765.1 |
| *Rhodobacter capsulatus* | NifH | WP_013066316.1 |
| *Chlorobaculum tepidum* | NifH | WP_010933198.1 |
| *Clostridium pasteurianum* | NifH | WP_003447877.1 |
| *Methanosarcina acetivorans* | NifH | WP_011023791.1 |
| *Rhodopseudomonas palustris* | AnfH | WP_011157001.1 |
| *Rhodobacter capsulatus* | AnfH | WP_013066329.1 |
| *Azotobacter vinelandii* | AnfH | P16269.1 |
| *Rhodopseudomonas palustris* | VnfH | WP_011156939.1 |
| *Azotobacter vinelandii* | VnfH | P15335.1 |
| *Methanococcus maripaludis* | NifH | WP_011170797.1 |


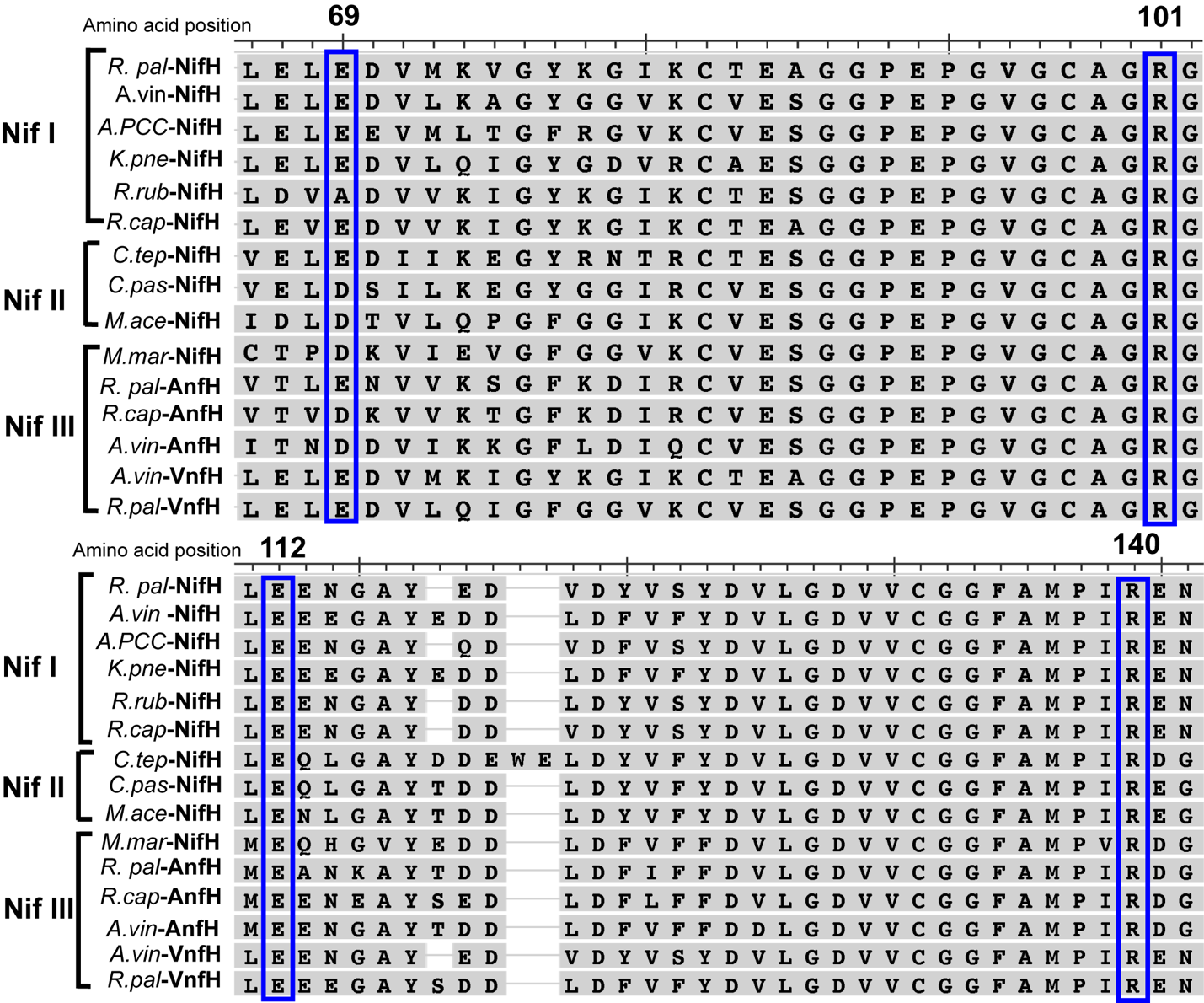


**Supplemental Fig. 1. Multiple amino acid sequence alignments of Fe protein homologs from Nif clades.** Charged residues including arginine (R) 101, glutamate (E) 112, and arginine (R) 140 are 100% conserved; residue glutamate (E) 69 is 70% conserved or replaced with mostly aspartate (D) among Fe proteins of Nif clades (Nif I, Nif II, and Nif III) [37]. COBALT (NCBI) [38] was used to align amino acid sequences of Nif, Vnf, or Anf homologs from selected model organisms including *R.pal*: *Rhodopseudomonas palustris*, *A.vin*: *Azotobacter vinelandii*, *K.pne*: *Klebsiella pneumoniae*, *R.rub*: *Rhodospirillum rubrum*, *R.cap*: *Rhodobactor capsulatus*, *C.tep*: *Chlorobaculum tepidum*, *C.pas*: *Clostridium pasteurianum*, *M.ace*: *Methanosarcina acetivorans, M.mar*: *Methanococcus maripaludis*.

**
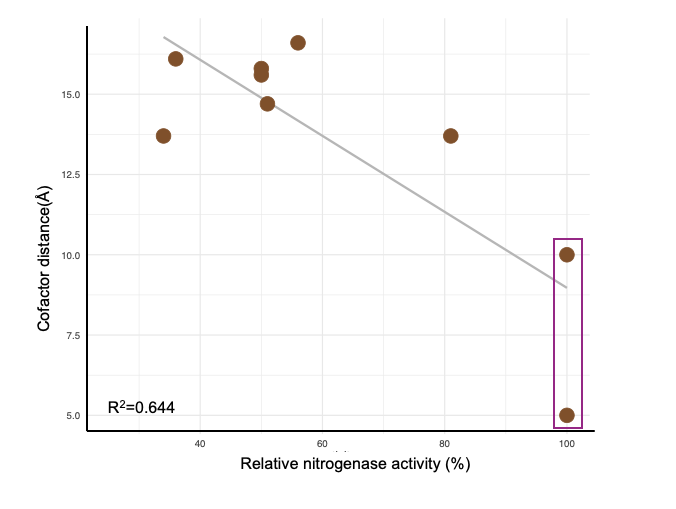
**

**Supplemental Fig. 2.** Positive correlation between the measured cofactor distance range and relative nitrogenase activity reported in Yang et al.; 2017. Boxed data points for nitrogen fixing ferredoxins and plant ferredoxins are without box.

**Supplemental table 5: 2[4Fe-4S] ferredoxins heterologously expressed in *R. palustris*.**

| **Organism** | **Ferredoxin name** | **NCBI accession number** | **PDB ID for template** |
| --- | --- | --- | --- |
| *Clostridium pasteurianum* | *Cp*Fd | P00195.2 | 1CLF[14] |
| *Thermotoga maritima* | *Tm*1175 | WP_004080188.1 | 1RGV[9] |
| *Chlorobaculum tepidum* | *Ct*FdII | WP_010932930.1 | 1RGV[9] |
| *Esherichia coli* | *Ec*Fd | WP_001196283.1 | 2ZVS[39] |
| *Thermotoga maritima* | *Tm*1815 | WP_004082361.1 | 2ZVS[39] |

**Supplemental table 6. Doubling times for heterologously expressed 2[4Fe-4S] ferredoxins in *R. palustris.***

| **Genotype** | **Doubling time (hours) in N_2_-fixing conditions^a^** | **Statistical significance compared to WT**  **(*p-*value)^b^** |
| --- | --- | --- |
| WT | 11 (0.1) | NS^d^ |
| ∆PEC | ND^c^ | ND |
| ∆PEC P_fer1_::*Cp*Fd | 12 (0.2) | NS |
| ∆PEC P_fer1_::*Tm*1175 | 31 (0.9) | 0.0019 |
| ∆PEC P_fer1_::*Ec*Fd | 85 (7.7) | 0.0034 |
| ∆PEC P_fer1_::*Ct*Fd | 89 (8.3) | 0.0038 |
| ∆PEC P_fer1_::*Tm*1815 | 129 (22) | 0.0094 |

^a^Values represent mean of three biological replicates with standard deviation shown in parentheses.

^b^Significance was determined using a one-way ANOVA followed by a Dunnett test with WT as a control.

^c^ND, not determined because this strain is unable to grow under these conditions.

^d^NS, not significant

**Supplemental Table 7: Strains, plasmids, and primer used for this study.**

| **Strains** | **Description** | **Reference** |
| --- | --- | --- |
| WT | CGA009 | [40] |
| In-frame deletion PEC | Deletion of electron carrier protein encoding genes – *fer1 (rpa4631)*, *fldA (rpa2117)*, *ferN (rpa4629)*, and *badB (rpa0062)* | This study |
| In-frame deletion PEC p*fer1*::*Ec* Fd | ∆PEC strain with *E. coli*Fd expressed at the *fer1* locus | This study |
| In-frame deletion PEC p*fer1*::Ctep Fd | ∆fer1ferNfldAbadB strain with *C. tepidum* Fd expressed at the *fer1* locus | This study |
| In-frame deletion PEC p*fer1*::Cpas Fd | ∆PEC strain with *C. pasteurianum* Fd expressed at the *fer1* locus | This study |
| In-frame deletion PEC p*fer1*::*Tm*1175 Fd | ∆PEC strain with *T. maritima* TM1175 Fd expressed at the *fer1* locus | This study |
| In-frame deletion PEC pfer1::*Tm*1815 | ∆PEC strain with *T. maritima* TM1815 Fd expressed at the *fer1* locus | This study |
| **Plasmid** | **Description, host strain, and selective marker** | **Reference** |
| p*fer1::*Cpas Fd | pJQ200Sk vector *C. pasteurianum* Fd with 25bp upstream and downstream of *fer1* in S17-1 *E. coli*, gentamycin 20µg/mL | This study |
| p*fer1::*Ctep Fd | pJQ200Sk vector *C. tepidum* Fd with 25bp upstream and downstream of *fer1* in S17-1 *E. coli*, Gentamycin 20µG/mL | This study |
| p*fer1::*Ec Fd | pJQ200Sk vector expressing *E. coli* Fd with 25bp upstream and downstream of *fer1* in S17-1 *E. coli*, Gentamycin 20µG/mL | This study |
| p*fer1::*TM1175 Fd | pJQ200Sk vector expressing *T. maritima* TM1175 Fd with 25bp upstream and downstream of *fer1* in S17-1 *E. coli*, Gentamycin 20µG/mL | This study |
| p*fer1::*TM1815 Fd | PJQ200Sk vector expressing *T. maritima* TM1815 Fd with 25bp upstream and downstream of *fer1* in S17-1 *E. coli*, Gentamycin 20µG/mL | This study |
| **Primer name** | **Primer sequence (5’ to 3’)** | **Reference** |
| pJQ200SK_vecF | CGCTGATCTCCGAAGCCGAGCACGGGCTCTAGAACTAGTGGATCCCCGG | [41] |
| pJQ200SK_vecR | TCGAATTCCTGCTCCTGCAAGACGCCTCCAGCTTTTGTTCCCTTTAGTGAGGG | [41] |
| Cp_usF | ATGGCCTACAAGATCGCCGACTCGTG | This study |
| Cp_dsR | TTACTCCTGGACCGGGGCGC | This study |
| CtFdII_usF | ATGGCCCTGTACATCACCGAGGAG | This study |
| CtFdII_dsR | TTAGCCCTGGACGATGCACTCGG | This study |
| Ec_usF | ATGGCCCTGCTGATCACCAAGAAG | This study |
| Ec_dsR | TTAGATCTTGTCGGCGTGGTGCATC | This study |
| Tm1175_usF | ATGGCCAAGAACTGGTACCCGGTC | This study |
| Tm1175_dsR | TTAGCCGTCGGCCGAGACCTC | This study |
| Tm1815_usF | ATGGCCGAGGCCAAGAACGCCCC | This study |
| Tm1815_dsR | TTACGGCTCCGGCTTGGTCTCGGTCTCC | This study |

**Supplementary references**

1. Fixen KR, Pal Chowdhury N, Martinez‐Perez M, et al (2018) The path of electron transfer to nitrogenase in a phototrophic alpha‐proteobacterium. *Environ Microbiol* 20:2500–2508. https://doi.org/10.1111/1462-2920.14262

2. Rey FE, Harwood CS (2010) FixK, a global regulator of microaerobic growth, controls photosynthesis in *Rhodopseudomonas palustris*. *Mol Microbiol* 75:1007–1020. https://doi.org/10.1111/j.1365-2958.2009.07037.x

3. Kostylev M, Otwell AE, Richardson RE, Suzuki Y (2015) Cloning should be simple: *Escherichia coli* DH5α-mediated assembly of multiple DNA fragments with short end homologies. *PLoS One* 10:e0137466. https://doi.org/10.1371/journal.pone.0137466

4. Simon R, Priefer U, Puhler A (1983) A broad host range mobilization system for *in vivo* genetic engineering: transposon mutagenesis in gram negative bacteria. *Nat Biotechnol* 1:784–791. https://doi.org/10.1038/nbt1183-784

5. Grote A, Hiller K, Scheer M, et al (2005) JCat: a novel tool to adapt codon usage of a target gene to its potential expression host. *Nuc Acids Res* 33:W526–W531. https://doi.org/10.1093/nar/gki376

6. Giastas P, Pinotsis N, Efthymiou G, et al (2006) The structure of the 2[4Fe–4S] ferredoxin from *Pseudomonas aeruginosa* at 1.32-Å resolution: comparison with other high-resolution structures of ferredoxins and contributing structural features to reduction potential values. *J Biol Inorg Chem* 11:445–458. https://doi.org/10.1007/s00775-006-0094-9

7. Lewis NM, Kisgeropoulos EC, Lubner CE, Fixen KR (2024) Characterization of ferredoxins involved in electron transfer pathways for nitrogen fixation implicates differences in electronic structure in tuning 2[4Fe 4S] Fd activity. *J Inorg Biochem* 254:112521. https://doi.org/10.1016/j.jinorgbio.2024.112521

8. Khan S, Ansari A, Brachi M, et al (2024) Structure, dynamics, and redox reactivity of an all-purpose flavodoxin. *J Biol Chem* 300:107122. https://doi.org/10.1016/j.jbc.2024.107122

9. Unciuleac M, Boll M, Warkentin E, Ermler U (2004) Crystallization of 4-hydroxybenzoyl-CoA reductase and the structure of its electron donor ferredoxin. *Acta Crystallogr D Biol Crystallogr* 60:388–391. https://doi.org/10.1107/S0907444903028506

10. Jacobson BL, Chae YK, Markley JL, et al (1993) Molecular structure of the oxidized, recombinant, heterocyst [2Fe-2S] (iron-sulfur) ferredoxin from *Anabaena* 7120 determined to 1.7-.ANG. resolution. *Biochemistry* 32:6788–6793. https://doi.org/10.1021/bi00077a033

11. Hurley JK, Weber-Main AM, Stankovich MT, et al (1997) Structure−function relationships in *Anabaena* ferredoxin: correlations between X-ray crystal structures, reduction potentials, and rate constants of electron transfer to ferredoxin: NADP^+^ reductase for site-specific ferredoxin mutants. *Biochemistry* 36:11100–11117. https://doi.org/10.1021/bi9709001

12. Alagaratnam S, van Pouderoyen G, Pijning T, et al (2005) A crystallographic study of cys69ala flavodoxin II from *Azotobacter vinelandii*: structural determinants of redox potential. *Protein Sci* 14:2284–2295. https://doi.org/10.1110/ps.051582605

13. Deistung J, Thorneley RNF (1986) Characterization of flavodoxin from *Azotobacter chroococcum* and comparison of its redox potentials with those of flavodoxins from *Azotobacter vinelandii* and *Kiebsiella pneumoniae* (*nifF*-gene product). *Biochem J* 239:69–75. https://doi.org/10.1042/bj2390069.

14. Bertini I, Donaire A, Feinberg BA, et al (1995) Solution Structure of the Oxidized 2[4Fe-4S] Ferredoxin from *Clostridium pasteurianum*. *Eur J Biochem* 232:192–205. https://doi.org/10.1111/j.1432-1033.1995.tb20799.x

15. Saeki K, Tokuda K, Fukuyama K, et al (1996) Site-specific mutagenesis of *Rhodobacter capsulatus* ferredoxin I, FdxN, that functions in nitrogen fixation. *J Biol Chem* 271:31399–31406. https://doi.org/10.1074/jbc.271.49.31399

16. Stout C, Stura E, McRee D (1998) Structure of *Azotobacter vinelandii* 7Fe ferredoxin at 1.35 Å resolution and determination of the [Fe-S] bonds with 0.01 Å accuracy. *J Mol Biol* 278:629–639

17. Yakunin AF, Gogotov IN (1983) Properties and regulation of synthesis of two ferredoxins from *Rhodopseudomonas capsulata*. *BBA Bioenerg* 725:298–308. https://doi.org/10.1016/0005-2728(83)90203-7

18. Pérez-Dorado I, Bortolotti A, Cortez N, Hermoso JA (2013) Structural and phylogenetic analysis of *Rhodobacter capsulatus* NifF: uncovering general features of nitrogen-fixation (*nif*)-flavodoxins. *Int J Mol Sci* 14:1152–1163

19. Kelley LA, Mezulis S, Yates CM, et al (2015) The Phyre2 web portal for protein modeling, prediction and analysis. *Nat Protoc* 10:845–858. https://doi.org/10.1038/nprot.2015.053

20. Zhang T, Zhang A, Bell SG, et al (2014) The structure of a novel electron-transfer ferredoxin from *Rhodopseudomonas palustri*s HaA2 which contains a histidine residue in its iron–sulfur cluster-binding motif. *Acta Crystallogr D Biol Crystallogr* 70:1453–1464. https://doi.org/10.1107/S139900471400474X

21. Dietrich HM, Righetto RD, Kumar A, et al (2022) Membrane-anchored HDCR nanowires drive hydrogen-powered CO_2_ fixation. *Nature* 607:823–830. https://doi.org/10.1038/s41586-022-04971-z

22. Hilge M, Gloor SM, Rypniewski W, et al (1998) High-resolution native and complex structures of thermostable β-mannanase from *Thermomonospora fusca* – substrate specificity in glycosyl hydrolase family 5. *Structure* 6:1433–1444. https://doi.org/10.1016/S0969-2126(98)00142-7

23. Morales R, Charon M-H, Hudry-Clergeon G, et al (1999) Refined X-ray structures of the oxidized, at 1.3 Å, and reduced, at 1.17 Å, [2Fe−2S] ferredoxin from the cyanobacterium *Anabaena* PCC7119 show redox-linked conformational changes. *Biochemistry* 38:15764–15773. https://doi.org/10.1021/bi991578s

24. Bes MT, Parisini E, Inda LA, et al (1999) Crystal structure determination at 1.4 Å resolution of ferredoxin from the green alga *Chlorella fusca*. *Structure* 7:1201-S2. https://doi.org/10.1016/S0969-2126(00)80054-4

25. Terauchi AM, Lu S-F, Zaffagnini M, et al (2009) Pattern of expression and substrate specificity of chloroplast ferredoxins from *Chlamydomonas reinhardtii*. *J Biol Chem* 284:25867–25878. https://doi.org/10.1074/jbc.M109.023622

26. Ikemizu S, Bando M, Sato T, et al (1994) Structure of [2Fe—2S] ferredoxin I from *Equisetum arvense* at 1.8 Å resolution. *Acta Crystallogr D Biol Crystallogr* 50:167–174. https://doi.org/10.1107/S0907444993009588.

27. Franke P, Freiberger S, Zhang L, Einsle O (2025) Conformational protection of molybdenum nitrogenase by Shethna protein II. *Nature* 998–1004. https://doi.org/10.1038/s41586-024-08355-3

28. Armengaud J, Meyer C, Jouanneau Y (1997) A [2Fe-2S] ferredoxin (FdVI) is essential for growth of the photosynthetic bacterium *Rhodobacter capsulatus*. *J Bacteriol* 179:3304–3309. https://doi.org/10.1128/jb.179.10.3304-3309.1997

29. Shinohara F, Kurisu G, Hanke G, et al (2017) Structural basis for the isotype-specific interactions of ferredoxin and ferredoxin: NADP^+^ oxidoreductase: an evolutionary switch between photosynthetic and heterotrophic assimilation. *Photosynth Res* 134:281–289. https://doi.org/10.1007/s11120-016-0331-1

30. Matsumura T, Kimata-Ariga Y, Sakakibara H, et al (1999) Complementary DNA cloning and characterization of ferredoxin localized in bundle-sheath cells of maize Leaves1. *Plant Physiol* 119:481–488. https://doi.org/10.1104/pp.119.2.481

31. Akashi T, Matsumura T, Ideguchi T, et al (1999) Comparison of the electrostatic binding sites on the surface of ferredoxin for two ferredoxin-dependent enzymes, ferredoxin-NADP*+* reductase and sulfite reductase. *J Biol Chem* 274:29399–29405. https://doi.org/10.1074/jbc.274.41.29399

32. Grinter R, Josts I, Mosbahi K, et al (2016) Structure of the bacterial plant-ferredoxin receptor FusA. *Nat Commun* 7:13308. https://doi.org/10.1038/ncomms13308

33. Hanke GT, Kimata-Ariga Y, Taniguchi I, Hase T (2004) A post genomic characterization of *Arabidopsis* ferredoxins. *Plant Physiol* 134:255–264. https://doi.org/10.1104/pp.103.032755

34. Shaheen S, Barrett KF, Subramanian S, et al (2020) Solution structure for an *Encephalitozoon cuniculi* adrenodoxin-like protein in the oxidized state. *Protein Sci* 29:809–817. https://doi.org/10.1002/pro.3818

35. Müller JJ, Hannemann F, Schiffler B, et al (2011) Structural and thermodynamic characterization of the adrenodoxin-like domain of the electron-transfer protein Etp1 from *Schizosaccharomyces pombe*. *J Inorg Biochem* 105:957–965. https://doi.org/10.1016/j.jinorgbio.2011.04.001

36. Kurisu G, Kusunoki M, Katoh E, et al (2001) Structure of the electron transfer complex between ferredoxin and ferredoxin-NADP+ reductase. *Nat Struct Mol Biol* 8:117–121. https://doi.org/10.1038/84097

37. Raymond J, Siefert JL, Staples CR, Blankenship RE (2004) The natural history of nitrogen fixation. *Mol Biol Evol* 21:541–554. https://doi.org/10.1093/molbev/msh047

38. Papadopoulos JS, Agarwala R (2007) COBALT: constraint-based alignment tool for multiple protein sequences. *Bioinformatics* 23:1073–1079

39. Saridakis E, Giastas P, Efthymiou G, et al (2009) Insight into the protein and solvent contributions to the reduction potentials of [4Fe-4S]^2+/+^ clusters: crystal structures of the *Allochromatium vinosum* ferredoxin variants C57A and V13G and the homologous *Escherichia coli* ferredoxin. *J Biol Inorg Chem* 14:783–799. https://doi.org/10.1007/s00775-009-0492-x

40. Huang JJ, Heiniger EK, McKinlay JB, Harwood CS (2010) Production of hydrogen gas from light and the inorganic electron donor thiosulfate by *Rhodopseudomonas palustris*. *Appl Environ Microbiol* 76:7717–7722. https://doi.org/10.1128/AEM.01143-10

41. Lewis NM, Sarne A, Fixen KR (2023) Evolving a new electron transfer pathway for nitrogen fixation uncovers an electron bifurcating-like enzyme involved in anaerobic aromatic compound degradation. *mBio* 14:e02881-22. https://doi.org/10.1128/mbio.02881-22
